# The effect of bone inhibitors on periosteum-guided cartilage regeneration

**DOI:** 10.1038/s41598-020-65448-5

**Published:** 2020-05-20

**Authors:** Hui-Yi Hsiao, Chao-Min Cheng, Shu-Wei Kao, Jia-Wei Liu, Chun-Shin Chang, Leila Harhaus, Jung-Ju Huang

**Affiliations:** 1 Division of Microsurgery Reconstructive Microsurgery, Department of Plastic and Reconstructive Surgery, Chang Gung Memorial Hospital, Chang Gung University, College of Medicine, Taoyuan, Taiwan; 2 Center for Tissue Engineering, Chang Gung Memorial Hospital, Taoyuan, Taiwan; 3 Institute of Biomedical Engineering, National Tsing Hua University, Hsinchu, Taiwan; 4 Department of Craniofacial Surgery, Department of Plastic and Reconstructive Surgery, Chang Gung Memorial Hospital, Chang Gung University, College of Medicine, Taoyuan, Taiwan; 5 Department of Plastic Surgery of Heidelberg University, BG Trauma center Ludwigshafen, Ludwigshafen, Germany

**Keywords:** Regenerative medicine, Tissue engineering

## Abstract

The regeneration capacity of knee cartilage can be enhanced by applying periosteal grafts, but this effect varies depending on the different sources of the periosteal grafts applied for cartilage formation. Tibia periosteum can be used to enhance cartilage repair. However, long-term analysis has not been conducted. The endochondral ossification capacity of tibia periosteum during cartilage repair also needs to be investigated. In this study, both vascularized and non-vascularized tibia periosteum grafts were studied to understand the relationship between tissue perfusion of the periosteum graft and the effects on cartilage regeneration and bone formation. Furthermore, anti-ossification reagents were added to evaluate the efficacy of the prevention of bone formation along with cartilage regeneration. A critical-size cartilage defect (4 × 4 mm) was created and was covered with an autologous tibia vascularized periosteal flap or with a non-vascularized tibia periosteum patch on the knee in the rabbit model. A portion of the vascularized periosteum group was also treated with the anti-osteogenic reagents Fulvestrant and IL1β to inhibit unwanted bone formation. Our results indicated that the vascularized periosteum significantly enhanced cartilage regeneration in the cartilage defect region in long-term treatment compared to the non-vascularized group. Furthermore, the addition of anti-osteogenic reagents to the vascularized periosteum group suppressed bone formation but also reduced the cartilage regeneration rate. Our study using vascularized autologous tissue to repair cartilage defects of the knee may lead to the modification of current treatment in regard to osteoarthritis knee repair.

## Introduction

Due to a lack of blood supply and a source of mesenchymal stem cells (MSCs), the self-repairing capacity of articular cartilage is limited^1^. As a result, injury of the articular cartilage is often irreversible, as seen in osteoarthritis of the elderly. To manage advanced osteoarthritis and patients’ quality of life, surgical treatments are often required, such as mosaicplasty or chondroplasty. Total knee replacement may ultimately be indicated with an artificial knee joint, which has possible drawbacks and complications, such as the requirements of anesthesia and surgery in the elderly, foreign body implantation and the potential risk of infection. The identification a new treatment strategy to enhance the repair capacity of the injured articular cartilage surface is in high clinical demand.

Periosteum was previously shown to contain chondrogenic and osteogenic capacity for chondrogenesis and osteogenesis^2–4^. In particular, vascularized periosteum is a great source for promoting osteogenesis and bone formation^5–8^. Different from current surgical treatments other than joint replacement, such as chondroplasty in which the treatments rely on residual cartilage, the use of the autologous periosteal graft may have the potential of the cartilage regeneration. Autologous periosteal graft has become an alternative treatment to repair articular cartilage^9^.

The chondrogenic factors released from periosteum, such as Transforming growth factor beta 1 (TGF-ß1), growth and differentiation factor-5 (GDF-5), bone morphogenetic protein-2 (BMP-2), and integrins, are required and sufficient to induce chondrogenesis^10^. However, using the vascularized periosteum for cartilage repair permits the risk of subsequent ossification. In addition, cartilage repair by non-vascularized periosteum mainly acted through endochondral mechanisms^11^. The result indicated that endochondral ossification appears to be favored when the periosteum graft is implemented as a non-vascularized graft^12^. This result is contrary to our previous study, in which an axial-patterned vascularized tibia periosteal flap was applied to repair cartilage defects of the knee in a rabbit model and showed promising cartilage repair 4 weeks after surgery in comparison to the non-periosteum group^13^. Even though the vascularized periosteum provided chondrogenic factors to promote chondrogenesis for cartilage repair, endochondral ossification was observed in the repaired region 8 weeks after the surgery in our follow-up experiments. A natural process of endochondral ossification includes a sequence of hypertrophy and death of chondrocytes, the subsequent invasion of blood vessels and osteoblasts in the extracellular matrix of cartilage and a resultant deposit of bone on the cartilage matrix^11^. Blood supply is believed to be a crucial factor for the ossification process by bringing osteoblasts to the cartilage. The source of the blood supply from the vascular pedicle of the periosteal flap might be a contributing factor. Considering the controversial effects of vascularity in the periosteum, the first goal of our study was to compare the cartilage regeneration capacity using the vascularized pedicled periosteal flap and a non-vascularized periosteal graft in long-term treatment.

Even though neocartilage regeneration was enhanced in our previous study, ossification on the regenerated cartilage was eventually observed. The type of ossification in the tibia starts from endochondral ossification, in which the ossification process starts with the formation of cartilage that is later converted into a calcified matrix, leading to bone formation^11^. Whether or not cartilage undergoes endochondral ossification is an event of major concern in the long run, even if chondrogenesis can be seen in early stage. According to the literature, the prevention of ossification during cartilage formation can likely be achieved by different mechanisms. Wang *et al*. observed the inhibition of osteogenic differentiation via estrogen deficiency by ovariectomizing rats, which activates the NFκB-pathway^14^. To reduce systemic side effects, we created an alternative method to induce topical estrogen deficiency by topically applying estrogen receptor (ESR) antagonists. Fulvestrant (Ful) is a new 7α-alkylsulfinyl analog of 17β-estradiol that is different in chemical structure from the nonsteroidal structures of tamoxifen, raloxifene and other SERMs. Ful binds, blocks, and accelerates degradation of the ER protein and leads to complete inhibition of estrogen signaling through the ER^15–17^.

Another strategy of inhibiting osteogenic differentiation is via the use of proinflammatory cytokines, which have been shown to inhibit osteogenic differentiation and bone formation^18–20^. Nuclear factor kappa B (NF-κB) is a master regulator of inflammation and host immune responses. NF-κB can be activated by proinflammatory cytokines, such as tumor necrotic factor (TNF), interleukin 1β (IL-1β), LPS, and viral DNA, in response to inflammatory disease and tissue injury^21–24^. IL1β, which is a cytokine produced by helper T cells, also potently inhibits MSC differentiation by activating IKK–NF-kB, indicating that immune cells can likely impair MSC-mediated bone regeneration and repair during inflammation^25^.

To avoid the natural process of ossification and maintain engineered cartilage, the present study was designed to investigate the possibilities of inhibiting the differentiation of periosteal stem cells for the production of osteocytes and osteoblasts. To this end, we applied Ful and IL1β to inhibit ossification during cartilage regeneration. We believe that preventing ossification during cartilage regeneration will be an important to promote successful clinical application of vascularized pedicled periosteal flaps in cartilage repair.

## Results

### Pedicled vascularized periosteal flap enhanced cartilage regeneration

**Figure 1:**
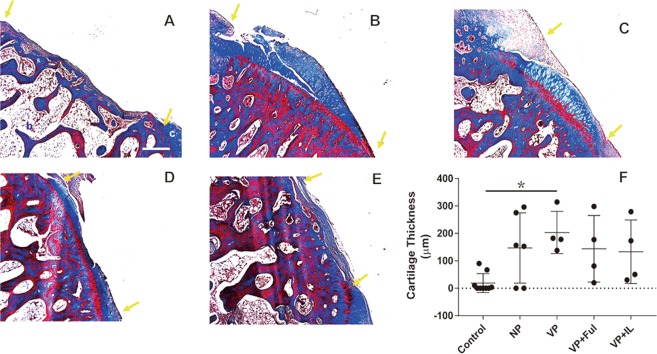
Histological analysis with Masson’s Trichome staining. The thickness of neo-cartilage in the (**A**) control group, (**B**) non-vascularized group, (**C**) vascularized group, (**D**) vascularized + Ful group and (**E**) vascularized + IL1β group. (**F**) The thickness of neo-cartilage was quantified by measuring the tissue thickness of neo-cartilage from the bone-cartilage junction to the superficial layer of regenerated tissue in the cartilage defect area. The area between two yellow arrows indicates the cartilage defect region. Sample size: n > 4. Scale bar = 1 mm. The results are expressed as the means ± SEM. Significant differences were determined by one-way ANOVA and the Tukey-Kramer post hoc test. *Indicates statistical significance (*p* < 0.05). Control: cartilage defect group. NP: cartilage defect covered with non-vascularized periosteum group. VP: cartilage defect covered with vascularized periosteum group. VP + Ful: cartilage defect covered with vascularized periosteum and treated with Fulvestrant. VP + IL-1β: cartilage defect covered with vascularized periosteum and treated with interleukin 1β. c: cartilage tissue; b: bone tissue.

In this study, the effect of the periosteum on neo-cartilage formation was evaluated in the rabbit knee defect model, and the effect of anti-ossification regimens was investigated. A total of 5 groups, each of which included the defect, with various treatments were included. After the animals were euthanized, the previous surgical areas were opened, and the previously transferred periosteal flaps or grafts (if any) were removed to expose the defect and examine the defect repair. The cartilage defects were considered without neo-cartilage regeneration when the bone tissue was exposed and no sign of cartilage formation could be identified. The cartilage defect of the control group remained, with only thin layered fibrous tissue covering the defect 8 weeks after surgeries (Fig. 1A); this confirmed that the defects were critical-sized. A similar type of regeneration with fibrous tissue was observed in the group with the defect repaired by non-vascularized periosteal flap coverage (Fig. 1B). Unlike the control or the non-vascularized periosteum flap groups, pedicled vascularized periosteal flaps enhanced cartilage repair, with the gross appearance of smooth neo-cartilage regeneration (Fig. 1C). Our next step was to investigate the effect of topical treatment using IL 1β or Ful to prevent ossification. To this end, the vascularized pedicled periosteal flap was selected to repair the defect, and the medications were given as described above. The gross appearance of the cartilage defect repair was less promising when compared to the healing observed in the repair of the vascularized pedicle periosteal group without the medication treatment. In these two groups that received the medication treatment, the defect region only showed partial cartilage regeneration and was occasionally accompanied by fibrous tissue (Fig. 1D,E).

### Vascularized periosteal flap but not medication enhanced neo-cartilage distribution microscopically

The neo-cartilage distribution was observed using Masson’s Trichome staining. The cartilage thickness was quantified by measuring the thickest area of the neo-cartilage tissue. The neo-cartilage thickness in the non-vascularized periosteum flap group tended to be higher in comparison to the control group, but this difference was not high enough to reach statistical significance (146.8 ± 52.26 μm vs. 19.02 ± 11.05 μm, respectively, *p* = 0.1) (Fig. 1F). The pedicled vascularized periosteal flap group showed significantly thicker neo-cartilage than the control group (203.2 ± 38.43 μm vs. 19.02 ± 11.05 μm, respectively, *p* = 0.026). The results indicated that the non-vascularized periosteum itself was able to regenerate neo-cartilage but was not sufficient for recovery of the whole defect region within 8 weeks. In the bone inhibitor treatment groups, Flu (10 µM) and IL1β (1 ng/mL) were applied to the cartilage defect region every two weeks. At the end point of the study, gross observation of the knee defects did not show any differences among all four groups. However, by adding Flu or IL1β to the vascularized periosteal flap group, the neo-cartilage formation was not enhanced. Both the Ful group and the IL1β group presented thicker neo-cartilage in comparison to the control group, but neither of these groups presented with higher neocartilage thickness than the vascularized pedicled periosteum flap group without medication (203.2 ± 38.43 μm vs. 141.1 ± 60.63 μm, *p* = 0.89; 203.2 ± 38.43 μm vs. 133.2 ± 57.98 μm, *p* = 0.82).

**Figure 2:**
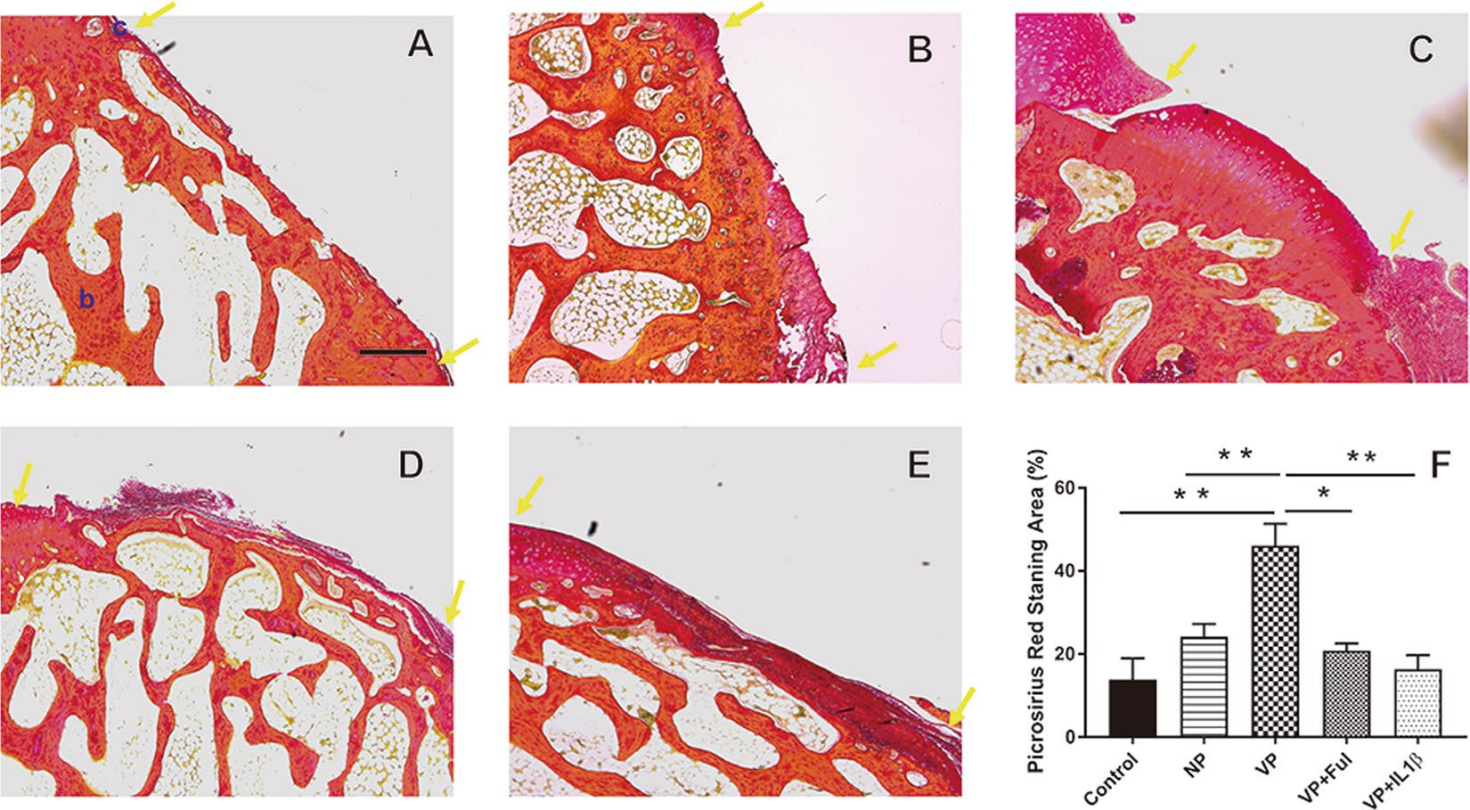
The collagen level of neo-cartilage tissue evaluated by Picrosirius Red assay. (**A**) control group, (**B**) non-vascularized group, (**C**) vascularized group, (**D**) vascularized + Ful group and (**E**) vascularized + IL1β group. The collagen level of cartilage was quantified by Picrosirius Red assay. The area between two yellow arrows indicates the cartilage defect region. c: cartilage tissue; b: bone tissue. Sample size: n > 4. Scale bar = 200 μm. The results are expressed as the means ± SEM. Significant differences were determined by one-way ANOVA and the Tukey-Kramer post hoc test. *Indicates statistical significance (*p* < 0.05). **Indicates statistical significance (*p* < 0.01).

After histological evaluation using Masson’s Trichome staining, Picosirius Red assay was used to measure the collagen level of the neo-cartilage^26^. In the control group, most of the specimens exhibited a discontinuous surface, with fibrous tissue covering the defect region (Fig. 2A). The tissue covering the defect region in the non-vascularized group was primarily comprised of fibrous cartilage tissue (dark red color), but fibrous cartilage growth was not sufficient to integrate with the neighboring cartilage tissue (Fig. 2B). Among the regenerated cartilage surfaces, the collagen level of the specimens in the vascularized periosteum group presented significantly higher levels in comparison to that of the specimens in the control and non-vascularized groups (46.14 ± 5.22% vs. 13.86 ± 5.13%, *p* < 0.0001; 46.14 ± 5.22% vs. 24.17 ± 3.03%, *p* = 0.006, respectively) (Fig. 2C,F). Although the neo-cartilage was grossly observed in the treatment group, the collagen level in the treatment groups was similar to the collagen level in the non-vascularized group; there was no significant difference (20.83 ± 1.74% vs. 24.17 ± 3.03%, *p* = 0.98; 16.4 ± 3.31% vs. 24.17 ± 3.03%, *p* = 0.73) (Fig. 2D,F). The defects treated with the bone inhibitor seem to hinder neo-cartilage formation.

### More mature cartilage presented in the vascularized periosteum group

**Figure 3:**
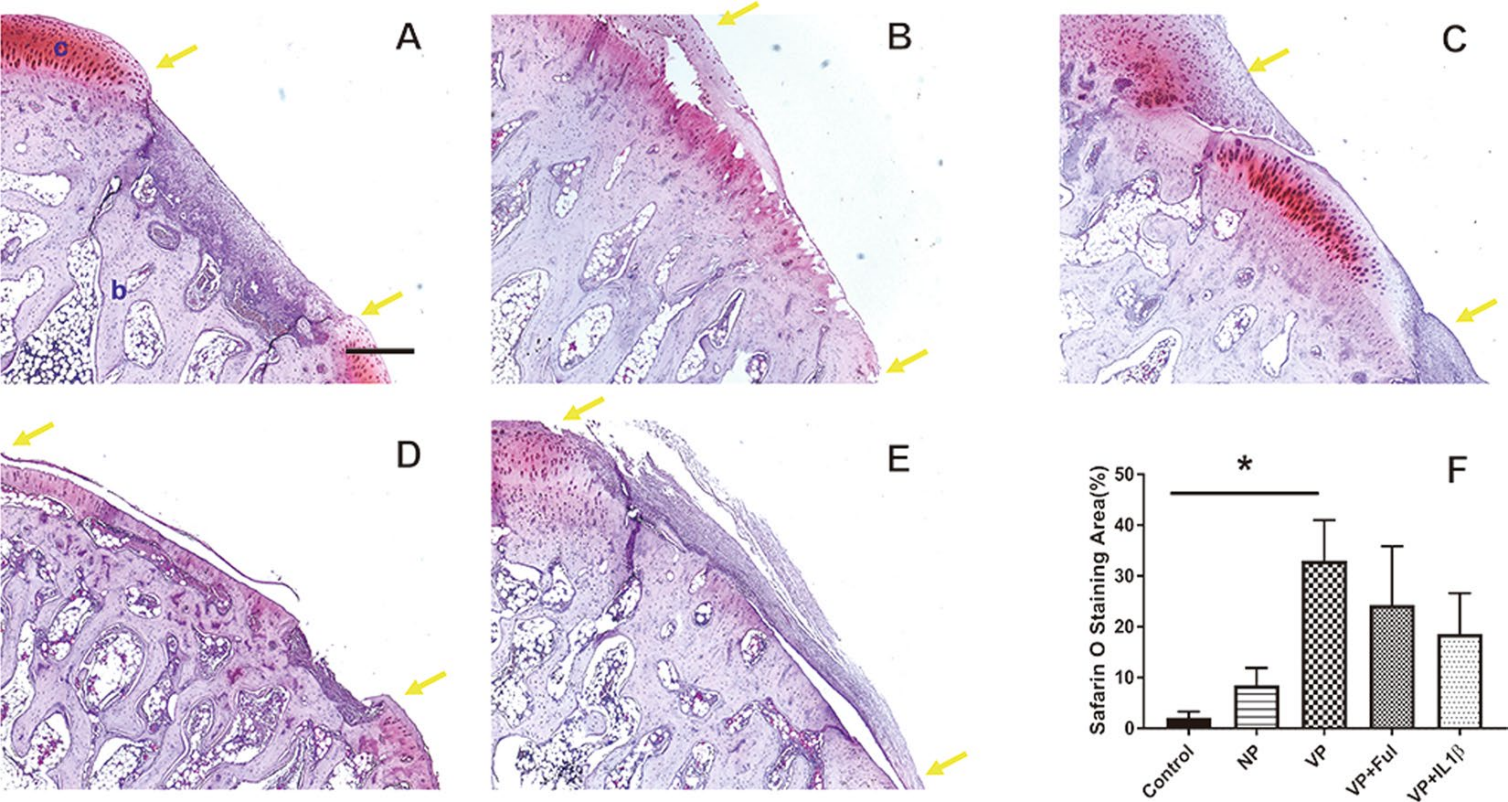
The neo-cartilage formation in the (**A**) control group, (**B**) non-vascularized group, (**C**) vascularized group, (**D**) vascularized + Ful group and (**E**) vascularized + IL1β group. (**F**) The cartilage content was quantified by Safranin O assay. The area between two yellow arrows indicates the cartilage defect region. c: cartilage tissue; b: bone tissue. Sample size: n > 4. Scale bar = 200 μm. The results are expressed as the means ± SEM. Significant differences were determined by one-way ANOVA and the Tukey-Kramer post hoc test. *Indicates statistical significance (*p* < 0.05).

**Figure 4:**
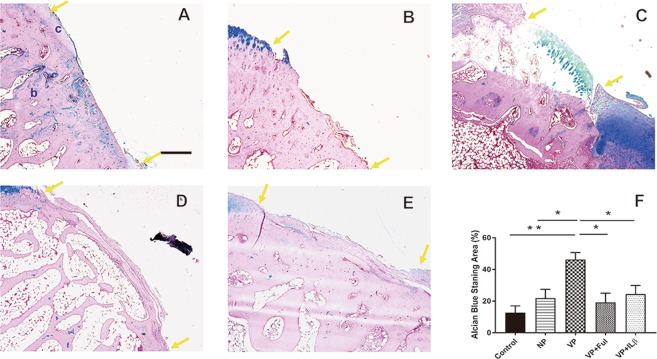
The expression of proteoglycan formation in the (**A**) control group, (**B**) non-vascularized group, (**C**) vascularized group, (**D**) vascularized + Ful group and (**E**) vascularized + IL1β group. (**F**) The cartilage content was quantified by alcian blue assay. The area between two yellow arrows indicates the cartilage defect region. Sample size: n > 4. Scale bar = 200 μm. c: cartilage tissue; b: bone tissue. The results are expressed as the means ± SEM. Significant differences were determined by one-way ANOVA and the Tukey-Kramer post hoc test. *Indicates statistical significance (*p* < 0.05).

A Safranin O assay was performed to identify specific patterns in the mature cartilage tissue (Fig. 3A–E). Stronger staining of Safranin O presented in the neo-cartilage of the vascularized group (Fig. 3C). Compared to the vascularized group, the non-vascularized group showed weaker Safranin O staining (Fig. 3B). Similarly, weaker Safranin O staining was also observed in the groups receiving Ful or IL1β. The mature cartilage tissue area was calculated by measuring the Safranin O staining. A larger area of mature cartilage was identified in the vascularized periosteum group compared to the control group (33.01 ± 7.98% vs. 2.09 ± 1.21%, *p* = 0.02). The groups with Ful or IL1β treatment generated a similar level of mature cartilage area as the non-vascularized group (24.32 ± 11.53% vs. 8.5 ± 3.4%, *p* = 0.60; 18.62 ± 7.99% vs. 8.5 ± 3.4%, *p* = 0.86). The neo-cartilage formation was further verified using the Alcian blue assay to detect the proteoglycan expression within the extracellular matrix of cartilage tissue^27^. The proteoglycan level of the vascularized group was significantly higher than those of the other groups (Fig. 4A–F). The content of proteoglycan in the group treated with bone inhibitors were similar to the content of proteoglycan in the non-vascularized group (21.80 ± 5.66% vs. 19.2 ± 5.80%, *p* = 0.99; 21.80 ± 5.66% vs. 24.43 ± 5.48%, *p* = 0.99).

**Figure 5:**
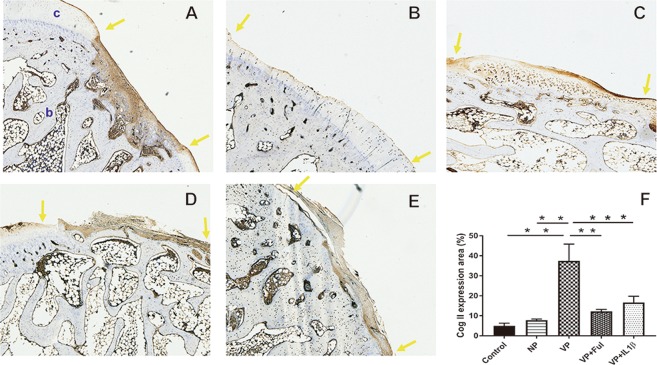
The expression of Collagen II in neo-cartilage tissue in the (**A**) control group, (**B**) non-vascularized group, (**C**) vascularized group, (**D**) vascularized + Ful group and (**E**) vascularized + IL1β group. (**F**) The expression level of collagen II was quantified. The area between two yellow arrows indicates the cartilage defect region. c: cartilage tissue; b: bone tissue. Sample size: n > 4. Scale bar = 200 μm. The results are expressed as the means ± SEM. Significant differences were determined by one-way ANOVA and the Tukey-Kramer post hoc test. *Indicates statistical significance (*p* < 0.05).

**Figure 6:**
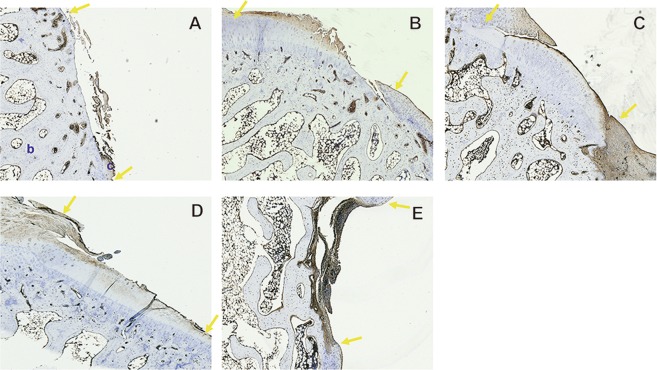
The expression of LPR4 in neo-cartilage tissue in the (**A**) control group, (**B**) nonvascularized group, (**C**) vascularized group, (**D**) vascularized + Ful group and (**E**) vascularized + IL1β group. (**F**). The area between two yellow arrows indicates the cartilage defect region. c: cartilage tissue; b: bone tissue. Sample size: n > 4. Scale bar = 200 μm.

The expression of collagen type II in articular cartilage was evaluated. In the control group, collagen II staining could be identified, but many of the stains were found in the bone tissue area within the defect region (Fig. 5A). The level of collagen II expression was greater in the VP group than in the NP and control groups (37.46 ± 8.33 vs. 7.81 ± 0.57%, *p* < 0.0001; 37.46 ± 8.33 vs. 4.98 ± 1.32%, *p* < 0.0001) (Fig. 5F). Agrin, a heparan sulfate proteoglycan, can promote cartilage differentiation through binding to the receptor of low-density lipoprotein receptor-related protein 4 (LRP4). To understand whether this periosteum-guided cartilage formation was dependent on argin, LRP4 expression was investigated. LRP4 expression was low and was more prominently located in the native cartilage neighboring the defect area in all the groups (Fig. 6A–E).

### Bone inhibitors successfully prevented endochondral ossification during cartilage regeneration

**Figure 7:**
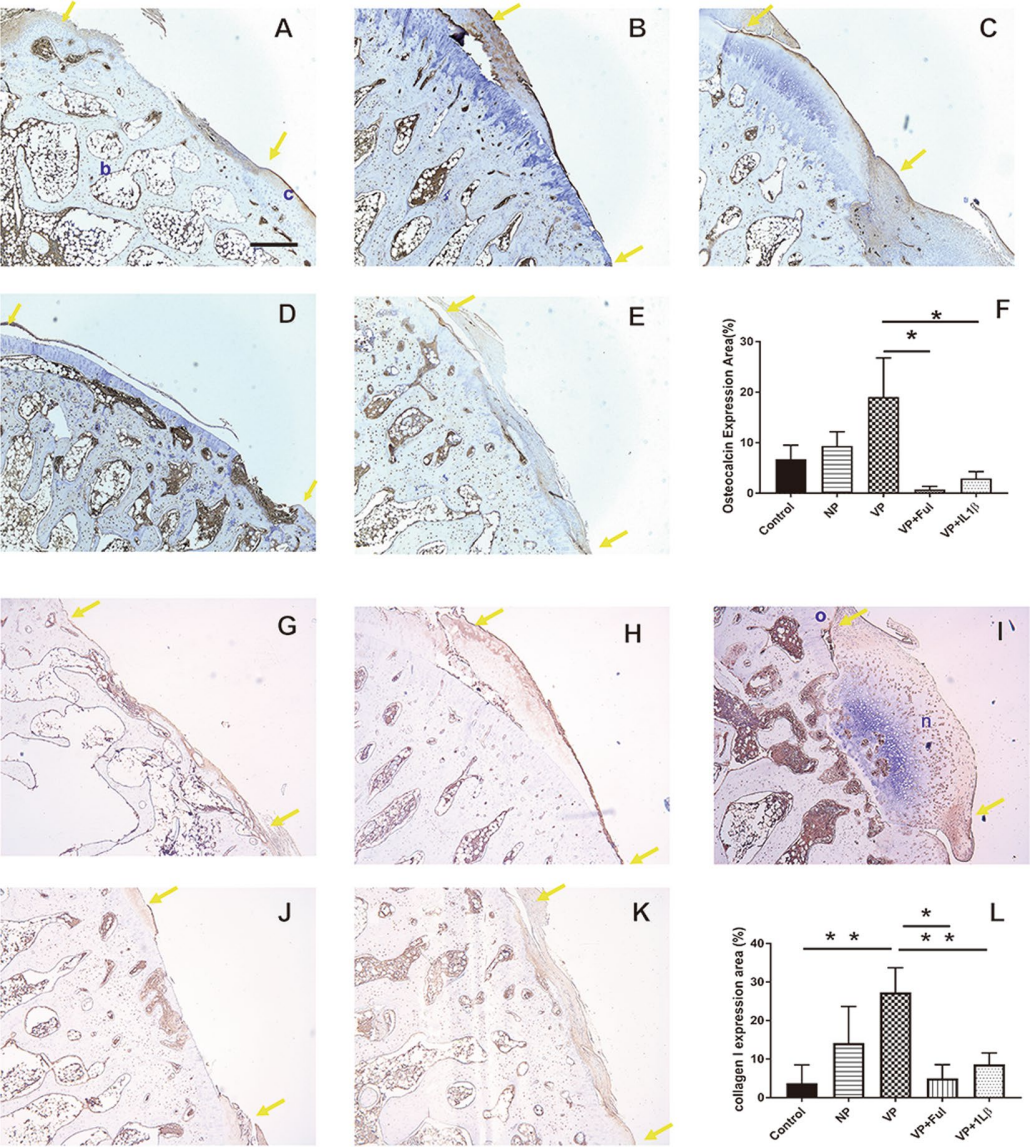
The detection of bone formation under periosteum treatment in the (**A**) control group, (**B**) nonvascularized group, (**C**) vascularized group, (**D**) vascularized + Ful group and (**E**) vascularized + IL1β group. (**F**) The osteocalcin expression that is indicative of bone was quantified. (**G**) control group, (**H**) nonvascularized group, (**I**) vascularized group, (**J**) vascularized + Ful group and (**K**) vascularized + IL1β group. (**L**) The collagen I expression that is indicative of bone was quantified. The area between two yellow arrows indicates the cartilage defect region. c: cartilage tissue; b: bone tissue. Sample size: n > 4. Scale bar = 200 μm. The results are expressed as the means ± SEM. Significant differences were determined by one-way ANOVA and the Tukey-Kramer post hoc test. *Indicates statistical significance (*p* < 0.05).

In our previous study, we used vascularized periosteum to successfully enhance cartilage repair^13^, but subsequent ossification was not preventable. To prevent ossification, Ful and IL1β were selected as bone inhibitors in the current study. Osteocalcin, which is a bone marker, was evaluated by staining to identify bone formation and to evaluate osteogenic activity under the different treatments. Osteocalcin only presented in the exposed bone tissue located in the cartilage defect region in the negative control group (Fig. 7A). Higher osteocalcin expression was identified in the non-vascularized and vascularized groups (Fig. 7B,C). Both Ful and IL1β successfully prevented ossification; there was a dramatic reduction in osteocalcin compared to the osteocalcin in the vascularized periosteum group (0.73 ± 0.64% vs. 19. 01 ± 7.75%, *p* = 0.01; 2.94 ± 1.3% vs. 19.01 ± 7.75%, *p* = 0.02) (Fig. 7D–F). Furthermore, the expression of collagen type I was also monitored. Collagen type I is known as the predominated collagen type in bone tissue^28^. The expression of collagen type I was significantly higher in vascularized group by compared to control group (27.3 ± 6.4% vs. 3.77 ± 2.1%, *p* < 0.001) (Fig. 7G–L). However, the groups with treatment of Ful and IL1β presented drastically reduction of collagen type I expression by compared to that of vascularized group (5.01 ± 1.44% vs. 27.3 ± 6.4%, *p* = 0.001; 3.77 ± 2.1% vs. 27.3 ± 6.4%, *p* = 0.01) (Fig. 7G–L). Those results indicate that the bone inhibitors were able to reduce bone formation. However, at the same time, the regeneration of cartilage was also compromised.

## Conclusion

Human and rabbit periosteum exhibit similarities in their structural organization comprising cambrium and fibrous layers^29,30^. The thickness of cambium layer both in rabbit and human periosteum were also decreased with age^31^. The rabbit has been reported as the smallest readily available laboratory mammal available allowing different surgical procedures to be performed in a reproducible manner^32^. Hence, the rabbit model was chosen as a relevant and feasible animal model for the study of periosteum’s regenerative capacity in cartilage repair. In the current study, our aims were to investigate the role of the inclusion of vascularity in the periosteum to enhance chondrogenesis and to determine whether or not supplementation with two bone inhibitors, Ful and IL1β, helps in reducing osteogenesis during the process of cartilage repair. Our results indicated that the control group without periosteum covering presented very low cartilage regeneration, which led to exposure of the bone tissue that was at the bottom of the cartilage defect region. The non-vascularized group demonstrated neo-cartilage formation that primarily comprised fibrous cartilage formation. Integration with neighboring cartilage was incomplete and resulted in a discontinuous or irregular cartilage surface. The vascularization that most likely supplied by peripheral circulation system to support the non-vascularized periosteum provides osteogenic factor or stem cells for cartilage regeneration. Unlike the non-vascularized periosteum group, the vascularized periosteum group presented complete neo-cartilage formation that filled the defect region. The integration with neighboring cartilage was continuously complete, and the surface of the regenerated cartilage was smooth. Our results indicated that the periosteum is important for promoting neo-cartilage formation, but complete results require vascularized transfer of the periosteum. Improved generation of mature cartilage was observed in the vascularized periosteum group in comparison to the non-vascularized periosteum group and to the groups with medication to prevent ossification.

Although enhancement of chondrogenesis was observed in the vascularized periosteum group, the richness of vascularity from the vascularized periosteum might further induce the tissue to undergo the process of ossification in cartilage repair. Of note, vascular invasion is a key step during endochondral ossification and is an absolute requirement to convert avascular cartilage into highly vascular bone^33,34^. The reduction of bone formation and hypertrophy of cartilage can be observed when vascular invasion into the cartilage is impaired^35^. With its osteogenic capacity, the use of periosteum to enhance chondrogenesis by delivering vascularity can significantly enhance ossification as well, which can be problematic. Our results also showed ossification in the vascularized periosteum group, confirming the presence of ossification as a natural process. Further research to modulate the vascularity and to convert the local environment into a relatively nonvascular status at a specific time point during chondrogenesis is necessary.

Due to the concern regarding the use of the vascularized periosteum to enhance the endochondral ossification process during long-term treatment, our next interest was to apply topical bone inhibitors to prevent ossification. Our investigation on the effects of the bone inhibitors Ful or IL1β on long-term cartilage regeneration revealed positive effects in terms of preventing ossification. We expected that the application of bone inhibitors might prolong cartilage formation by inhibiting endochondral ossification in the cartilage phase. Osteocalcin expression in the group with the bone inhibitor was significantly reduced compared to that in the vascularized group, which suggests that the process of bone formation was inhibited. However, the regenerated cartilage thickness was also reduced after treatment with the bone inhibitors. These results indicated that the bone inhibitors affect both cartilage and bone formation.

During the process of bone formation, cartilage acts as a scaffold for osteoblast differentiation and the deposition of bone matrix. Cartilage formation is a major process of bone formation. Neo-cartilage formation progresses through three phases: proliferation, development, and maturation^11^. Chondrocytes begin to proliferate between days 3 and 14, type II collagen increases, the matrix is synthesized between days 14 and 28, and cartilage maturation occurs in the subsequent days. To prevent further ossification, we treated the cartilage defect with anti-bone reagents every two weeks following defect creation to the end of study. We selected bone inhibitors to target the NF-kB pathway. Our results showed that both chondrogenesis and ossification were suppressed. Although the NF-kB pathway is important for osteogenic differentiation, recent studies have also shown its relationship to chondrogenesis^18–20^. The role of NF-kB in mediating growth plate chondrogenesis has been reported. The expression of NF-kB in the growth plate facilitates bone growth by inducing chondrocyte proliferation and differentiation^36,37^. Han and colleagues demonstrated that NF-kB is one of the critical factors that mediate IL1β-induced suppression of chondrogenic potential in human synovial fluid-derived stem cells^38^. Taken together, the use of Ful and IL1β in our study is likely not only to prevent osteogenic differentiation but also to interfere with chondrogenesis, which is the first step of ossification. Although there have been conflicting data regarding the effect of Ful and IL1β on chondrogenesis, our application of both anti-bone reagents interfered with chondrogenesis and led to the suppression of neo-cartilage formation.

Our results demonstrate that the vascularized periosteum promotes neo-cartilage formation in long-term treatment and promotes significant repair in a created defect region. The further long-term supply of blood and nutrients from the vascularized periosteum does appear to induce osteogenesis, which is a primary and preliminary concern. On the other hand, we found that adding anti-osteogenic reagents resulted in the suppression of neo-cartilage formation. In summary, the vascularized periosteum itself is sufficient to enhance neo-cartilage formation and promote defect repair. However, the vascularized periosteum also likely promotes endochondral ossification to form unwanted bone. The addition of a validated dosage of bone inhibitor or modulation of periosteal vasculature may offer alternative approaches for clinical repair. Further studies will be required to clarify dosage requirements and to elucidate underlying mechanisms of these approaches. Further studies will be required to clarify dosage requirements and to elucidate underlying mechanisms of these approaches. Additionally, our study of the use of vascularized autologous tissue in repairing cartilage defects of the knee may lead to the modification of current treatments in regard to osteoarthritis knee repair. Our results may impact the clinical application, the development of naturally derived biomaterials, the engineering of cartilage tissue, and regenerative medicine.

## Materials and Methods

### Animals and surgical procedures

All animal use protocols were reviewed and approved by the institutional Animal Care and Use Committee (IACUC) of Chang Gung Memorial Hospital (IACUC: 2013090401). All experimental protocols were performed according to the animal research guidelines and regulations of Chang Gung Memorial Hospital. Eight 3-month-old New Zealand white rabbits weighing approximately 3 kg each were used for the animal study. The selection of New Zealand white rabbits for the animal study was based on two reasons: 1) the biomechanical properties of the rabbit knee are similar to the human knee, and 2) the rabbit model is well-established in the field of joint cartilage research. A total of 32 defects were created on the 16 femoral condyles of the 8 rabbits.

**Figure 8:**
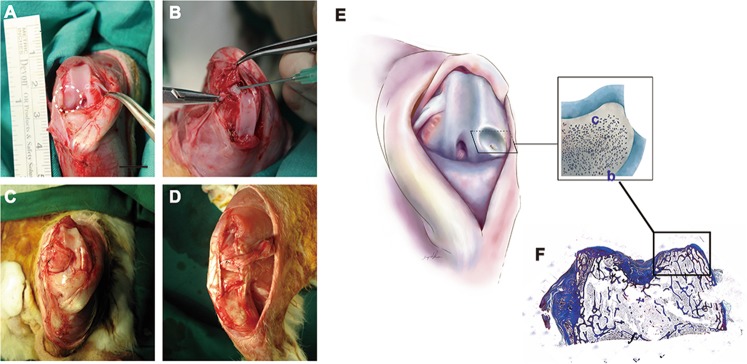
Knee defect rabbit model. (**A**) Bilateral cartilage defects were created with a rotating saw. (**B**) Periosteum was fixed on the defect cartilage area with TISSEEL (Baxter AG, Vienna) before being stitched onto the cartilage. (**C**) Non-vascularized periosteum group. (**D**) Vascularized periosteum group. (**E**) The schematic image of knee defect model. The inset image represented the cross section of defect region. The histology images throughout this study were presented in this view. c: cartilage tissue; b: bone tissue.

The anesthesia of the rabbits was achieved through intramuscular injection of a narcosis mixture of Zoletil^©^ (Virbac, Philippines) and Rompum^©^ (Xylazine Hydrochloride 23.32 mg/mL) (Bayer, Germany) at a ratio of 1:1 and a dose of 2.3 mL for a 3.0 kg rabbit for each injection. After anesthesia, the hair of the hind limb of interest was shaved, and the skin was sterilized by scrubbing using polyvidone iodine and covered with sterile covers. The surgical procedure of defect creation was described in our previous work^13^. Briefly, a longitudinal incision along the medial parapatellar line and ventral tibia was performed. The subcutaneous dissection was conducted, and a parapatellar incision of the medial capsule of the patella was made to localize the patella. The patella was then dislocated laterally to expose the knee joint and its overlying articular cartilage. A full-thickness cartilage defect of 4 × 4 mm was created using a rotating saw in the lateral and medial femoral condyle of both legs of the rabbit (Fig. 8A). Care was taken to avoid subchondral bone injury, as confirmed by the complete absence of bleeding. With this technique, 4 defects per animal could be achieved for our various analyses.

In the same leg as the knee cartilage defect, the rabbit autologous tibia periosteal flap, sized 3 × 15 mm, was harvested based on the saphenous artery and its venae comittantes with a perivascular tissue cuff, according to a previous description^13^. Special care was taken regarding the precise preparation and separation of the periosteal tissue from the bone while harvesting the periosteal flaps to ensure the inclusion of chondrocyte precursor cells, which are located in the cambium layer. The flap was harvested and fixed into the lesion site using a 6-0 transosseous ethilon suture and was further secured with single drops of Tissucol Duo S© two-component fibrinogen tissue glue (Fig. 8B). For the transfer of the non-vascularized periosteum patch, the 3 × 15 mm tibia periosteal flap without the saphenous artery attached was harvested and sutured to the knee defect area (Fig. 8C). The patella was repositioned after the flap transfer, the incision as closed with a layer of a 4-0 absorbable suture (PDS) (Johnson & Johnson, USA) at the capsular joint, and the superficial fascia and skin was sutured with 4-0 nylon materials (Johnson & Johnson, USA). With the pedicled flap design, a free rotation arc of the flap was created and allowed the germinal layer to face the joint cavity (Fig. 8D). The flap was fixed into the cartilage defect with the same technique. The cartoon image was used to present the defect region (Fig. 8E). The inset image represented the cross section of defect region. The histology images throughout this study were presented in this view. The overview of Masson’s trichrome cartilage image indicated the relative location of defect region in knee area (Fig. 8F).

#### Experimental design

In the study, a total of 5 groups were included, and each had at least 4 biological replicates.

1. The cartilage defect without treatment, i.e., the negative control (lateral condyles, left and right) (n = 9).
2. The cartilage defect with a non-vascularized periosteum patch (lateral condyle, right leg) and vascularized periosteal flap (medial condyle, right leg) (n = 8).
3. The cartilage defect with a vascularized periosteal flap (medial condyle, right leg) (n = 5).
4. The cartilage defect with a vascularized periosteal flap and treated with Fulvestrant (n = 5).
5. The cartilage defect with a vascularized periosteal flap and treated with IL 1β (n = 5).

Two ossification inhibitors, Ful and IL1β, were used in this study. The medications were given by injection into the knee defect region every two weeks until the end of the study. Dosing of the medications was based on previous reports. The reduction of the mineralization of Ful was reported to be dose-dependent as shown by the reduction of the ALP activity of mature osteoprecursor cultures^39^. A statistically significant difference was found at the concentration of 10 µM. Therefore, we selected 10 µM as the concentration in our study. A significant effect of cytokines in suppressing the osteoblast development of MSCs was reported by Lacey *et al*.^25^. The suppression was found at both the differentiation and proliferation levels. Murine IL-1β was applied at concentrations of 0.001–1 ng/mL, and the effect was observed even at the lowest level of 0.1 ng/mL (in cell culture). We chose a concentration of 1 ng/mL in our study to confirm the effective concentration for the prevention of mineralization.

## Euthanasia and specimen collection

The rabbits were sacrificed 8 weeks after surgery. The rabbits were euthanized by an overdose injection of lidocaine, and the limbs were prepared. During preparation, the ventral tibia diaphysis harvesting defects were examined macroscopically in terms of infection or possible partial necrosis of the cortical tibia bone due to reduced blood supply. Macroscopic appearances of the defect sites were examined and photographed. Visually acceptable repairs were considered when the repair was smooth, with firm repair tissue that filled the defects. The femur condyles were harvested, and the specimens were subjected to histological analysis.

## Histology Analysis and Evaluation

After harvesting, the specimens were fixed for histological examination in 4% buffered formalin. To examine both sides of the regenerative tissues attached to the native cartilage, each grafted area was dissected along the frontal plane. The specimens were decalcified in 10% nitric acid for a minimum of 2 weeks, dehydrated and embedded in paraffin according to routine methods, sectioned, and processed using Masson’s Trichome staining. The regeneration of the newly formed cartilage tissue started from bone tissue and grew gradually and outwardly. By compared with neighboring old cartilage tissue, the thickness of the newly formed cartilage tissue was comparatively thinner. The border of new-old cartilage tissue was identified based on the tissue structural consistency, defect filling rate and the condition of superficial layer of defect^40^. Another evaluation point was based on the morphology of different layers of mature cartilage to determine the newly formed cartilage. The cartilage content was detected by Safranin O analysis, Picrosirius Red assay and Alcian blue assay using standard protocols. By the presented staining, the presence of Safranin O, Picrosirus Red assay, Alcian blue assay and Osteocalcin expression were calculated by the formula listed below: osteocalcin (or alkaline phosphatase) expression area (%) = [staining area within defect area/total defect area].” The sections were digitally imaged using an Axio Scope A1 microscope (Carl, Zeiss, Germany). For each view area (5x objective, 1.4 mm × 1 mm area), the percentage of expression areas of all the cartilage markers were analyzed by Image-Pro Premier 9.0 software (Media Cybernetics, Inc.). Five images were randomly selected and quantified. There are four knee tissues included in each group and all of them were carefully evaluated. Two slides of each tissue had been used for histology analysis.

### Statistics analysis

The quantitative data were analyzed and compared using Prism 7 statistical software (GraphPad Software, Inc. USA) by one-way ANOVA and post hoc Tukey tests. The statistical error α goal was 5% and values with a difference of *p* < 0.05 were considered significant.
